# Self-medication practices among pregnant women in Ethiopia

**DOI:** 10.1186/s40545-023-00584-7

**Published:** 2023-06-19

**Authors:** Fentaw Girmaw, Ashenafi Kibret Sendekie, Betelhem Mesfin, Abebe Tarekegn Kassaw

**Affiliations:** 1 Department of Pharmacy, College of Health Science, Woldia University, Woldia, Ethiopia; 2 Department of Clinical Pharmacy, College of Medicines and Health Sciences, University of Gondar, Gondar, Ethiopia; 3 Department of Adult Health Nursing, School of Nursing, College of Health Science, Woldia University, Woldia, Ethiopia

**Keywords:** Self-medication, Pregnant women, Self-medication practice, Ethiopia

## Abstract

**Background:**

Self-medication is a worldwide issue that requires special attention due to the potentially harmful effects it can have not only on pregnant women but also on the fetus.

**Objectives:**

This study assessed the magnitude of self-medication practice and associated factors among pregnant women following antenatal care (ANC) in primary healthcare settings in the North Wollo Zone of Ethiopia.

**Methods:**

An institutional-based cross-sectional study was conducted on 395 pregnant mothers who attended ANC follow-up in selected health centers in the North Wollo Zone of Ethiopia from April 20 to May 20, 2021. A multi-stage sampling method was employed to enroll participants. A face-to-face structured interview was conducted to collect the data. A logistic regression analysis was used to determine the factors associated with self-medication practice. A *p* value < 0.05 at the 95% confidence level was considered statistically significant.

**Results:**

Out of a total of 444 participants approached, 395 (89%) participated in the study. Of these, 44.6% reported practicing self-medication during the current pregnancy. Age < 35 (AOR = 2.18, 95% CI 1.02–9.15; *p* = 0.032), rural residence (AOR = 3.01, 95% CI 1.43–10.19; *p* = 0.017), and previous medication use (AOR = 5.02, 95% CI 1.24–12.93; *p* = 0.015) were found to have a significant association with self-medication practice.

**Conclusion:**

Self-medication was highly prevalent among pregnant women in the study setting and result indicates need for critical action. Younger rural women with a history of self-medication use should be provided counselling to find a prescription medication, and measures are needed to minimize self-medication related harm in pregnant women.

**Supplementary Information:**

The online version contains supplementary material available at 10.1186/s40545-023-00584-7.

## Background

Pregnancy is a dynamic process characterized by enormous physiological changes that result in a variety of pregnancy-related symptoms such as headache, nausea, vomiting, and edema, which may lead to self-medication [1–4]. Self-medication is the use of medications to treat diseases or symptoms that one has self-diagnosed, as well as the intermittent or ongoing use of medications without a medical prescription [2, 5]. Self-medication is common among pregnant mothers in many developing countries. They self-medicate by using over-the-counter (OTC) drugs, prescribed medications, or herbal products. Self-medication with non-prescription OTC drugs available in pharmacies and drug stores has recently grown in popularity [6–8].

Self-medication is a major health and socioeconomic issue all over the world. The prevalence of self-medication varies greatly between developed and developing countries due to differences in sociocultural context and economic status [9, 10]. Because of the easy availability of a wide range of medicines, widespread poverty, and inadequate healthcare systems and facilities, the incidence of self-medication is particularly high in sub-Saharan Africa [11–13]. Several over-the-counter medications can be harmful during pregnancy. It is critical to assess the adverse effects of medications prescribed for various disease conditions during pregnancy on the mother and fetus. As a result, in most cases, the drugs of choice for treating conditions in pregnant and non-pregnant mothers are distinct [14]. According to various studies conducted among pregnant mothers, factors such as education level, income, access to medicines, knowledge, time, and age are associated with self-medication practice [15–17]. In Ethiopia, a hospital-based study of pregnant women at Jimma University's specialized hospital found that the prevalence of self-medication was 20.1% [16]. A previous finding in Mekelle hospital revealed that 9.5% and 2% of patients, respectively, self-medicate with herbal and modern medicines [17].

Inappropriate self-medication could have serious consequences and pose a global challenge, particularly among pregnant women and the elderly ages [18–22]. Self-medication, in fact, increases the risk of dependence, abuse, adverse drug reactions, misdiagnosis, incorrect therapy selection, and incorrect dosage forms [21, 22]. Ethiopia has one of the highest rates of maternal and newborn morbidity and mortality in the world. According to a federal ministry of health report, maternal mortality is estimated to be 673 deaths per 100,000 live births. Direct obstetric complications are responsible for 85% of these deaths [23]. Self-medication during pregnancy causes birth defects, miscarriages, and allergic diseases. Protecting the mother's and fetus's health by reducing problems associated with self-medication is critical [24–26]. Managing self-medication use during pregnancy could go a long way towards reducing maternal and child mortality due to drug and herb abuse.

In Ethiopia, some existing studies reported findings from hospitals and referral settings in major cities [3, 16, 17, 26–28]. However, the findings may differ from those obtained in rural and primary care settings due to differences in access to health facilities, differences in client awareness and perception, differences in regulatory body involvement, and other sociodemographic and clinical evidence. Thus, assessing self-medication practice among pregnant mothers in rural and primary care settings is critical for determining an appropriate intervention strategy. As a result, the purpose of this study was to determine the magnitude of self-medication and associated factors among pregnant mothers in selected primary health centers in the North Wollo Zone of Ethiopia. The study also assessed the reasons for and sources of self-medication among pregnant women in the study area.

## Methods

### Study design and setting

An institutional-based cross-sectional study was conducted in the ANC unit of selected health centers in Ethiopia's North Wollo Zone from April 20 to May 20, 2021. North Wollo has a population of 1,675,732 people, of whom 841,217 are men and 834,515 are women; 1,473,639 (87.94%) are rural residents. There are 65 primary healthcare centers (33 rural and 32 woreda towns) in the North Wollo Zone. The study was conducted at 13 primary health centers that were selected randomly by a lottery method.

### Study participants and eligibility criteria

Pregnant mothers who had received ANC follow-up at the selected primary health centers during the study period were included in the study. However, those who could not respond to the interview because of a serious illness and those who refused to participate were excluded from the study.

### Sample size determination and sampling procedures

The sample size was calculated using a single population proportion formula by considering the following assumptions: *n* = *p* (1 – *p*) * *Z*^2^/*W*^2^; *p* = 0.731, a population distribution taken from a study done in Hossana, South Ethiopia, on the prevalence of herbal medicines during pregnancy [26]; *W* = 0.05 of marginal error at the 95% confidence level (*Z* = 1.96). Then, by adding a 10% non-response rate and the design effect of 2, the total sample size required was 444.

**Fig. 1:**
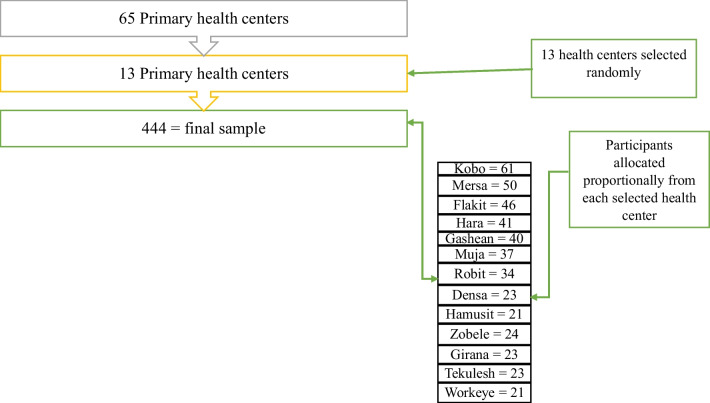
Systematic presentation of sampling approach to assess self-medication practice among pregnant women in the health centers of North Wollo Zone of Ethiopia

To select the study participants, a multi-stage sampling technique was implemented. Initially, 13 primary health centers were selected randomly. Then, using the one month previous ANC follow-up records of the health centers, a proportional allocation of participants was considered for the selected health centers (Fig. 1). The study participants were enrolled in the study using a systematic random sampling technique in each health center, with the first participant selected by lottery as the starting point. Using a sampling frame, eligible mothers were approached until the sample was maintained.

### Data collection instruments, procedures, and quality control

The data were collected through a face-to-face interview using a structured questionnaire that was adapted from a previous study and relevant literature [3, 26–31]. The adapted questionnaire was tailored to the local context and research objectives. The instrument consisted of three parts (Additional file 1). The first section consists of baseline sociodemographic and obstetric variables. The second consisted of a question regarding past and current self-medication practices and the reasons for and sources of medications. The third section consisted of the types of drugs used by the respondents.

To ensure the quality of the data collection instrument, experts in the field validated it for content and face validity. Furthermore, after the questionnaire was developed, a pre-test on 5% of the sample size was conducted at other similar health facilities nearby (Wuchale and Woldia health centers). Modifications were made in terms of clarifying and making long sentences concise, correcting ambiguous words, and correcting sequence. The feedback from the pre-test ensured the questions' clarity, wording, logical sequence, and skip patterns. Then, data collectors were trained on the study instrument and data collection procedure for one day before beginning actual data collection. Thirteen midwives were hired to collect data. The data collectors approached participants when they were at the health center for ANC follow-up. First, participants were asked for their voluntary participation and informed about the purpose, methodology, and ethical aspects of the procedure by the data collectors and principal investigator. Then, after consent was gained, participants were interviewed, and their responses were filled in the questionnaire. The interview took about 10–15 min on average. During data collection, the filled-out questionnaire was checked for its cleanliness, completeness, and clarity. To ensure the quality of the findings, the principal investigator checked for incompleteness, edited in the field and office, coded, and cleaned. Incomplete questionnaires were rejected and not considered for the analysis.

### Data entry and statistical analysis

Data were checked for completeness and consistency, then edited, coded, and entered into EpiData before being exported to the statistical software SPSS Window version 25 for analysis. A *Q*–*Q* plot and histogram were used to examine the data's normal distribution. Continuous variables were described using the mean and standard deviation, while categorical variables were presented using frequency and percentage. The association between self-medication practice among pregnant mothers and other independent variables was also investigated using logistic regression analysis. Variables with a *p* value of < 0.2 in the univariable analysis were investigated further in the multivariable analysis to identify potential variables associated with self-medication practice in pregnant women. An adjusted odds ratio with a *p* value of < 0.05 at the 95% confidence level was considered statistically significant.

## Results

### Sociodemographic and obstetrics characteristics of participants

A total of 395 pregnant women were included in the study, with an 89% response rate. The average age of the study participants was 26.8. ± 2.7, and 366 (92.7%) were under the age of 35. 93.4% (369) of those polled were married. Around 37.7% (149) of pregnant women in this study were unable to read and write. Approximately two-fifths (38.7%, 153 women) had a parity greater than or equal to two, 45.1% (178) had only their first ANC visit, 70.6% (279 women) were multigravida, and 43.3% (171) were pregnant in the second trimester (Table 1).

**Table 1:** Baseline sociodemographic and obstetrics variables of pregnant women (*N* = 395)

Variables	Category	Frequency	Percentage (%)
Age in years (mean ± SD)		26.8 ± 2.7	
Occupation	Farmer	178	45.1
	Merchant	81	20.5
	Government employer	62	15.7
	Housewife	74	18.7
Marital status	Married	369	93.4
	Not married	26	6.6
Education level	Unable to read and write	149	37.7
	Primary education	123	31.1
	Secondary education	54	13.7
	College and above	69	17.5
Residency	Rural	176	44.0.6
	Woreda town	219	55.4
Distance from health centers	≤ 5 km	105	26.6
	> 5 km	290	73.4
Parity	Nulliparity	134	33.9
	Para one	108	27.3
	≥ Para two	153	38.7
ANC attendance	First ANC visit	178	45.1
	ANC visit ≥ 2 times	217	54.9
Gravidity	Primigravida	116	29.4
	Multi-gravida	279	70.6
Stage of pregnancy	1st trimester	61	15.4
	2nd trimester	171	43.3
	3rd trimester	163	41.3

### Self-medication practice

**Fig. 2:**
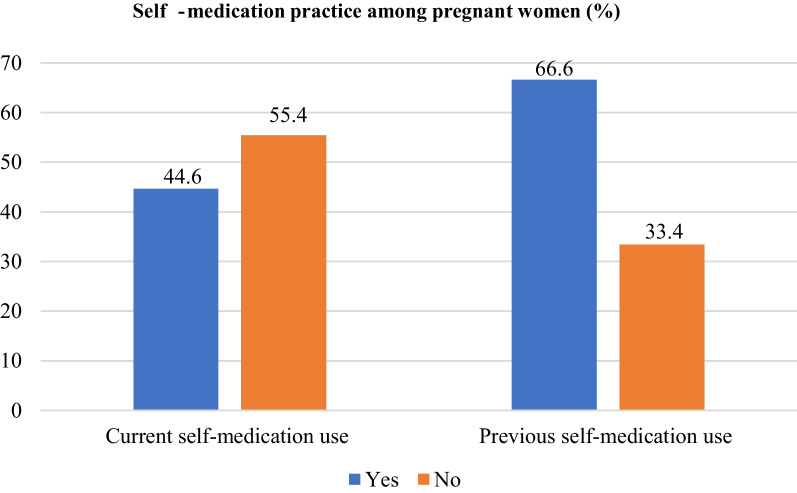
Prevalence of self-medication use among pregnant women (*N* = 395)

Around 44.6% (176) of the study participants reported using self-medications either with modern or herbal medicine in the current pregnancy, resulting in the prevalence of current self-medication practice among pregnant women being found to be 44.6% (95% CI: 36.6–53.3%); whereas two-thirds (66.6%, 263) of the respondents reported to have used self-medication in the past (Fig. 2).

### Reasons and sources of self-medications

The main reasons for self-medication were ease of access (47.7%, 84), followed by the belief that disease was not serious (40.3%, 71). Regarding medication sources, family and friends were the most common (35.2%, 62). Approximately 10% of pregnant women used left-over medications, while 14.8% obtained medications from their neighbors (Table 2).

**Table 2:** Reasons and source of self-medications among pregnant women (*N* = 176)

	Frequency	Percentage (%)
*Reasons of self-medications*		
Easily accessible	84	47.7
Disease not serious	71	40.3
Time saving	12	6.8
High cost of visiting health service	5	2.9
Long waiting time for health services	4	2.3
*Sources of medications*		
Family and friends	62	35.2
Pharmacy/drug shop	37	21.0
Neighbors	26	14.8
Traditional healers	23	13.1
Left-over medicines from any person	18	10.2
Left-over medicine from themselves	10	5.7

### Medications used for self-medication practice

Antibiotics (31.2%, 55) and analgesics (21.6%, 38) were the most commonly used medications for self-medication by pregnant women in the current study. Herbal medicines were used by approximately 13.1% (23) of the respondents (Table 3).

**Table 3:** Class of medicines used among pregnant women (*N* = 176)

Class of medicine used	Frequency	Percentage (%)
Antibiotics	55	31.2
Analgesics	38	21.6
Anthelmintic	29	16.5
Herbs	23	13.1
Gastrointestinal medicines	20	11.4
Others*	11	6.2

Others*, minerals and vitamins, antimalarial, antiemetics and anti-vomiting

### Association between self-medication and other independent variables

Multivariate logistic analysis revealed that only age, residence, and previous medication use were statistically significant with self-medication practice. Taking all other variables constant, the odds of self-medication use were roughly twice as high among those aged less than 35 compared to those aged 35 and above (AOR = 2.18, 95% CI: 1.62–9.15, *p* = 0.032). Similarly, mothers who lived in rural areas were approximately three times more likely to self-medicate than those who lived in Woreda towns (AOR = 3.01, 95% CI: 1.43–10.19, *p* = 0.017). Furthermore, mothers who had previously used medications been more likely to use self-medication than mothers who had not previously used medication (AOR = 5.02, 95% CI: 1.24–12.93, *p* = 0.015) **(**Table 4**).**

**Table 4:** Association of self-medication practice and other variables among pregnant women

Variables	Self-medication use		Univariable	Multivariable	
	Yes	No	COR (95% CI)	AOR (95% CI)	*P*-value
*Age*					
< 35	134	118	2.74 (1.25–5.99)	2.18 (1.62–9.15)	0.032*
≥ 35	42	101	1.0	1.0	
*Residence*					
Rural	115	71	3.604 (2.35–6.59)	3.01 (1.43–10.19)	0.017*
Town	71	158	1.0	1.0	
*Occupation*					
Farmer	109	69	3.733 (0.918–10.73)	2.54 (0.73–11.08)	0.067
Merchant	26	55	1.118 (0.64–4.79)	0.812 (0.251–3.17)	
Employee	19	4	11.23 (1.07–21.915)	5.035 (0.87–20.35)	
Housewife	22	52	1.0	1.0	
*Stage of pregnancy*					
1st trimester	41	20	3.613 (0.831–9.73)	1.78 (0.56–5.340)	0.321
2nd trimester	76	95	1.41 (0.371–3.04)	0.97 (0.034–5.321)	
3rd trimester	59	104	1.0	1.0	
*Previous medication use*					
Yes	163	100	11.9 (2.54–31.07)	5.02 (1.24–12.93)	0.015*
No	13	119	1.0		
*Distance from health center*					
≥ 5 km	170	120	2.34 (1.68–4.61)	1.37 (0.56–6.031)	0.135
< 5 km	6	99	1.0	1.0	

COR, crude odds ratio; AOR, adjusted odds ratio; *P-value significant (< 0.05)

## Discussion

This study assessed the prevalence and associated factors of self-medication among pregnant women attending antenatal care in selected health centers in the North Wollo Zone, Ethiopia. The study found that 44.6% (95% CI: 36.6–53.3%) of pregnant women practice self-medication in their current pregnancy. Ease of access to medications (47.7%) and the belief that disease was not serious (40.3%) were the most common reasons for self-medication. On the other hand, family and friends (35.2%) were the most common sources of self-medication practices. Antibiotics (31.2%) were the most commonly used medications for self-medication by pregnant women. Younger age, rural residence, and previous medication use were found to have a significantly positive association with self-medication practice.

The current findings revealed that the prevalence of self-medication was 44.6%. The magnitude noted in this study was greater than that observed in other studies [3, 29–31]. This might be because of the low accessibility of prescribed medications and health facilities in rural districts and woredas. In addition, differences in healthcare systems in terms of regulatory bodies involvement are another important factor. Furthermore, the current study included both modern and herbal medicine use among pregnant women, indicating that traditional medicine is a highly available source of healthcare in rural areas where health facilities are lacking [32]. However, the current finding was lower than other studies in a different part of Ethiopia [26–28] and the study conducted in Egypt [33]. The discrepancy might be because of the differences in populations related to sociodemographic factors. These earlier studies were conducted among pregnant mothers who visited hospitals and urban residents. As compared with the current study, there might be a difference in awareness, exposures, and experiences with self-medication.

The study also revealed that easy access to medicines and herbs were a major reason for self-medication practice. This finding was correlated with previous reports done in different areas [21, 34]. About 40.3% of the study participants reported that they practice self-medication because they believed their disease state was not serious. The low severity of the symptoms of illness was also frequently reported in the previous study [8, 35]. Different studies reported that familiarity with medicines and financial constraints [36], convenience and cost [37, 38], mildness of illness and cost [39], and saving time and ease of accessibility were the major reasons for self-medication. Thus, the current finding is in line with these different studies conducted earlier, and the finding may indicate the need for interventions for these frequently reported reasons for self-medication.

A significant proportion of pregnant women obtained medications from family and friends. This is in agreement with previous studies in Ethiopia and Kenya [28, 40]. The possible reason might be poor knowledge of self-medication use by family and friends. Surprisingly, in line with an earlier study, a significant proportion of women also used a left-over medication [41]. This may indicate a need for critical action to ensure the safe use of medications among pregnant mothers. The creation of awareness in the community regarding medication use might be important. In addition, regular ANC follow-up and counselling on the use of medications for pregnant mothers are crucial.

Antibiotics (31.2%), analgesics (21.6%), and anthelmintics (16.5%) were the three most commonly used drug classes among pregnant women, according to our findings. Similar research has been conducted in Addis Abeba, the southwest region of Ethiopia, and the Democratic Republic of the Congo [42–44]. This could be due to regulatory bodies' ineffective regulatory mechanisms in terms of controlling the dispensing and use of prescription medication. In the case of antibiotics, this leads to the emergence of antimicrobial drug resistance, which is a major concern because it can lead to higher treatment costs and negative drug effects. Additionally, in line with earlier studies, a significant proportion of pregnant mothers used herbal products, which might be dangerous for the fetus since some of the herbal products are known for their emmenagogue, mutagenic, and abortifacient effects [41, 45]. Therefore, health education is important for pregnant women and the community about the risks of self-medication. In addition, taking special measures to control frequently misused medications is critical.

The current study also demonstrated independent factors associated with self-medication practice in pregnant mothers. Younger age, rural residence, and history of self-medication were independent predictor variables for self-medication practice. In this study, pregnant mothers with an age less than 35 were more likely to use self-medication as compared to those who were greater than or equal to 35 years old. The finding implicates that providing counseling for younger pregnant women is important. However, this result was contrary to a study conducted at Nekemet Referral Hospital that showed pregnant women over the age of 40 were more likely to self-medicate [30]. The difference might be due to variations in the sociocultural context of the two study areas.

Consistent with an earlier study [41], pregnant mothers from rural districts were more likely to use self-medication than those from woreda towns. This could be because prescribed medications are inaccessible due to inadequate healthcare facilities and are inaccessible to pregnant women in rural areas compared with urban women. Another possible reason will be that they are less aware of the risks associated with self-medication. In addition, they are worried about the long waiting times and costs associated with visiting health centers. Respondents also stated that they had easy access to self-medications and used them due to the time savings and high cost of visiting a healthcare center. Another possible reason may be that self-prescribed herbal medicines might be used as a source of healthcare in rural areas. As a result of the findings, it is possible that an intervention focused on raising public awareness and ensuring regulatory involvement to ensure the safe and effective use of medications in pregnant women should be implemented. Thus, the findings indicate that interventions aiming at raising public awareness and ensuring regulatory involvement in order to ensure the safe and effective use of medications in pregnant women, with a focus on rural areas, should be implemented.

Among those respondents, two-thirds had prior self-medication experience, which was significantly associated with self-medication practice during pregnancy. This might be due to a lack of awareness among pregnant women about the use of drugs and/or herbs, as well as poor counselling from healthcare providers. This was discovered to be similar to the research done in Jimma, Nekemet, and Addis Abeba [16, 30, 42], as well as in Iran, the Democratic Republic of the Congo, and Nigeria [15, 43, 46]. One possible explanation is that people are unaware of the dangers of self-medication. The finding may indicate that strict intervention from healthcare providers, regulatory bodies, and health bureaus is required.

Generally, the current study highlighted the magnitude of self-medication use among pregnant women in rural areas, which is different from urban settings. It also assessed the reasons, sources, and associated factors of self-medication that need strict interventions and measures to ensure safe use of medications in pregnant women. An awareness-building campaign and health education for pregnant mothers as well as the general population, focusing on rural areas, need to be advocated. In addition, a strict regulatory body's involvement in the control of medication misuse and malpractice is necessary. Recommendations and guidelines from policymakers and programme designers that ANC providers can use to reduce the risk of self-medication use during pregnancy are important. Health care providers also need to be engaged in the promotion of safe use of medication for pregnant mothers.

### Limitation of the study

The study did not consider pregnant women who were at home during the study period. Since the study was cross-sectional, the findings cannot be used to infer a cause–effect relationship. Recall bias from previous experience may also be a limitation, and data collection confusion may also have an impact on the study's outcome. Therefore, prospective observational studies using a larger population-based study will be welcomed for future research.

## Conclusion

In this study, the use of self-medication among pregnant women in selected health centers was highly prevalent. It is critical to raise awareness among younger rural pregnant women with previous self-medication practices to reduce self-medication related harm during pregnancy.

## Supplementary Information

**Additional file 1.** Data collection instrument used in collecting the data to assess self-medication practice among pregnant mothers.

## Data Availability

The datasets generated and/or analyzed during the current study are available from the corresponding author on reasonable request.
