# Correction: Self-medication practices among pregnant women in Ethiopia

**DOI:** 10.1186/s40545-023-00600-w

**Published:** 2023-07-27

**Authors:** Fentaw Girmaw, Ashenaf Kibret Sendekie, Betelhem Mesfin, Abebe Tarekegn Kassaw

**Affiliations:** 1 Department of Pharmacy, College of Health Science, Woldia University, Woldia, Ethiopia; 2 Department of Clinical Pharmacy, College of Medicines and Health Sciences, University of Gondar, Gondar, Ethiopia; 3 Department of Adult Health Nursing, School of Nursing, College of Health Science, Woldia University, Woldia, Ethiopia

**Correction** **:** **Journal of Pharmaceutical Policy and Practice (2023) 16:74** 10.1186/s40545-023-00584-7

Following publication of the original article [1], it was reported that there was an error in Fig. 2. The correct Fig. 2 is given in this Correction article and the original article [1] has been updated.

**Fig. 2:**
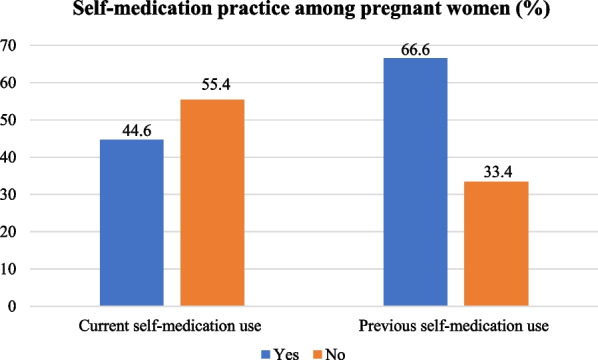
Prevalence of self-medication use among pregnant women (*N* = 395)
