# Supplementary material for: Self-medication practices among pregnant women in Ethiopia
Source: J Pharm Policy Pract. 2023 Jun 19;16:74. doi: 10.1186/s40545-023-00584-7 (PMC10278325; doi:10.1186/s40545-023-00584-7)
Supplement: Supplementary file 1 — Additional file 1. Data collection instrument used in collecting the data to assess self-medication practice among pregnant mothers. [file 40545_2023_584_MOESM1_ESM.docx]

**Informed consent and Data collection tools**

1. **Informed Consent form**

Dear participant,

We are from Woldia University research team, and we would like to kindly request your consent to participate on the study. The aim of this study is to assess “**Self-medication practice and associated factors among pregnant women having antenatal follow-up in primary health centers in the North Wollo Zone of Ethiopia”**. This is a cross-sectional study; the questioner comprises of questions regarding your socio-demographics information, obstetrics characteristics, medications that used as a self-medication practice. This questionnaire will hardly take your 10-15 minutes and all the information we obtain will remain strictly confidential and your answer and name will never be revealed. Your participation is greatly important of the research; however, we assure you that it is totally a voluntary participation and feel free to refuse or to withdraw at any point in the study.

Do you agree to participate in this study? 1. Yes ---- 2. No ----

If yes, please ready for interview.

**Part I. Sociodemographic, Obstetrics and related varaibles of the participants**

Table 1. Sociodemographic and obstetrics variables of pregnant women

| **Variables** | **Category** |
| --- | --- |
| Medical chart (Code) |  |
| Health center |  |
| Age in years | --- |
| Occupation | 1. Farmer 2. Merchant 3. Government employer 4. House wife 5. Other |
| Marital status | 1. Married 2. Not married 3. Divorced 4. Widowed |
| Education level | 1. Unable to read and write 2. Primary education 3. Secondary education 4. College and above |
| Residency | 1. Rural 2. Woreda town |
| Distance from health centers | 1. ≤ 5 km  2. > 5km |
| Parity | 1. Nulliparity 2. Para one 3. Para two 4. Para three 5. ≥ para four |
| ANC attendance | 1. First ANC visit 2. ANC visit ≥ 2 times |
| Gravidity | 1. Primigravida 2. Multi-gravida |
| Stage of pregnancy | 1. 1st trimester 2. 2^nd^ trimester 3. 3^rd^ trimester |

**Part II. Self-medication practice, reasons and sources of medications**

Table 2. Previous and current self-medication practice, reasons, and source of medications among pregnant women

| Varaibles |  |
| --- | --- |
| Current self-medication practice | 1. Yes 2. No |
| Previous self-medication use | 1. Yes 2. No |
| Reasons of self-medications | 1. Ease of accessibility 2. Disease not serious 3. Time saving 4. High cost of visiting health service 5. Long waiting time for health services 6. Other |
| Sources of medications | 1. Family and friends 2. Pharmacy/drug shop 3. Neighbors 4. Traditional healers 5. Left-over medicines 6. Themselves 7. Other |

**Part III. Class of medicines used as self-medication**

**Table 3.** Class of medicines used among pregnant women (N =176)

| **Varaibles** | **Category** |
| --- | --- |
| Medicines | 1. Antibiotics 2. Analgesics 3. Anthelmintic 4. Herbal medicine 5. Gastrointestinal medicines (proton pump inhibitors, antacids, H2 blockers, 6. Others if any ... |

Thank you for your participation!
